# Adapting Fabric Phase Sorptive Extraction as an Innovative Multitool for Sample Transfer and Extraction in Pharmacokinetic Analysis Followed by LC-MS Determination of Levofloxacin in Plasma Samples

**DOI:** 10.1021/acsomega.3c09519

**Published:** 2024-04-15

**Authors:** Yasemin Ekin Dolaksız, Mustafa Sinan Kaynak, Abuzar Kabir, Kenneth G. Furton, Mustafa Çelebier

**Affiliations:** † Faculty of Pharmacy, Department of Analytical Chemistry, Hacettepe University, 06230 Ankara, Turkiye; ‡ Faculty of Pharmacy, Department of Pharmaceutical Technology, Anadolu University, 26460 Eskişehir, Turkiye; § International Forensic Research Institute, Department of Chemistry and Biochemistry, Florida International University, 11200 SW 8th St., Miami, Florida 33199, United States

## Abstract

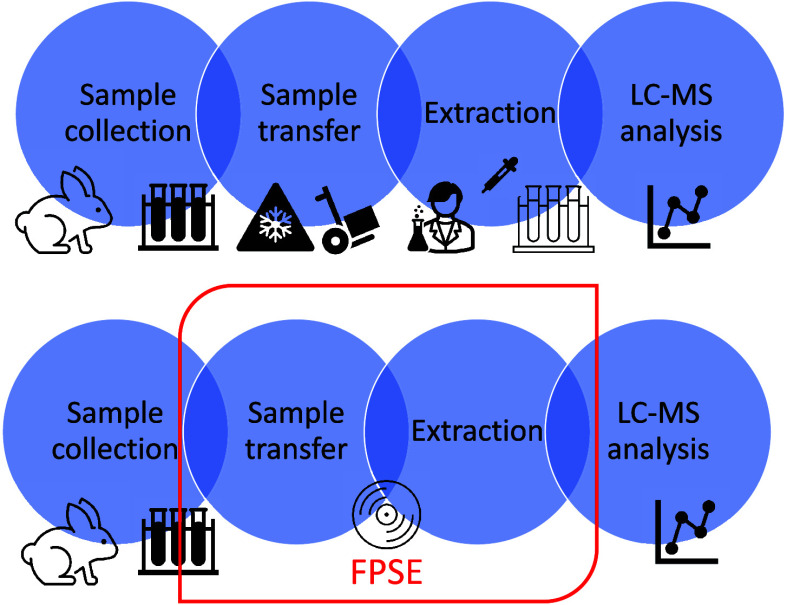

Fabric phase sorptive extraction (FPSE) is a simple microextraction technique that allows analytes to be rescued from matrix components while using a small volume of samples to analyze complex biological systems. This study used FPSE as a microextraction tool and a sample storage and transfer device. Levofloxacin as a model molecule was applied intravenously (IV) to New Zealand male rabbits. The samples were simultaneously extracted by using FPSE and protein precipitation methods. The final solutions were analyzed using LC-MS equipped with an ACE C18 LC Column (150 mm × 4.6 mm, 5 μm) at 25 °C employed in isocratic elution mode using solution A (0.1% formic acid in water)/solution B (0.1% formic acid in acetonitrile) (80:20, v/v). The total analysis time was less than 15 min. The developed method was validated using the ICH M10 bioanalytical method validation and study sample analysis guidelines. The results obtained using FPSE were statistically identical to those obtained using protein precipitation. The plasma samples applied onto FPSE (10 μL onto 1.0 cm × 1.0 cm Biofluid Sampler) were stored in three different temperatures [refrigerator (2–8 °C), at ambient temperature (20 ± 5 °C), and in the stability cabinet (40 °C, 75% humidity)] and three different storage conditions (Eppendorf tubes, plastic containers, and straw paper envelopes). Levofloxacin in plasma samples adsorbed by FPSE biofluid sampler remained stable at 2–8 °C in Eppendorf tubes for at least 1 week. This study showed that FPSE could be used as a sample storage and transfer device for pharmacokinetic applications that need to work with small sample volumes and discard aggressive cold chains to store and transfer the plasma samples.

## 1 Introduction

Active pharmaceutical substances are extracted from biological matrices by liquid–liquid extraction (LLE) or solid-phase extraction (SPE) in routine preclinical or clinical applications.^1^ The liquid–liquid extraction process conflicts with the demand for Green Analytical Chemistry principles due to the large volume of organic solvents that it consumes and the harmful effects of these solvents. Using complex extraction techniques in pharmaceutical analysis still plays an important role. Some extraction techniques are used to preconcentrate samples.^2^ Among existing sample preparation technologies, solid-phase extraction (SPE) is suitable for the simultaneous enrichment of various compounds from complex matrices.^3^ In solid-phase extraction, reproducible results can inevitably not be obtained due to the differences between the batches of sorbents. However, unsatisfactory sensitivity and time-consuming and costly operation procedures lead researchers to apply novel sample preparation techniques like microdialysis (MD), solid-phase microextraction (SPME), and microfluidic devices to improve the sensitivity, simplify the sample preparation steps, and deal with other challenges.^4^ Unlike these separation techniques, fabric phase sorptive extraction (FPSE), developed by Kabir and Furton in 2014, has attracted noticeable attention recently due to its ease of use, reproducibility, and compliance with Green Analytical Chemistry principles.^5,6^ Techniques developed in sample preparation in recent years require the analytical workflow and devices to be operated in accordance with the principles of Green Analytical Chemistry (GAC) and to be operated under minimal conditions.^7^

FPSE is a simple and rapid sample prepreparation process that allows extracting the target analytes directly from the unmodified sample matrices and successfully eliminates many steps typically involved in the classical sample preparation workflow that is carried out to eliminate the detrimental effect of the analysis of the complex sample matrix (protein digestion for biological materials, solvent removal, placebo removal for pharmaceutical preparations, etc.). FPSE helps analyze many components without the need to modify the matrix.^8^ FPSE has demonstrated its successful applications in a broad range of analytes from diverse sample matrices.^5^

The FPSE membrane consists of a layer of natural or synthetic fabric. This membrane is coated with a thin film of sol–gel organic–inorganic hybrid sorbent. The sol–gel sorbent has high chemical, thermal, and solvent impermeability and is chemically bonded to the fabric substrate.^9^ It selectively separated the substances to be analyzed from different sample matrices. By applying the sample directly to the membrane, the sol–gel sorbent coated on the membrane surface interacts with the analyte, and rapid separation and preconcentration of the target analytes to the FPSE membrane occurs. In this part, either the matrix interferents are selectively separated by the sol–gel sorbent, or the analyte(s) are attached to the membrane surface. Subsequently, the analyte(s) attached to the surface are selectively separated. In both ways, it is possible to selectively obtain the analyte within the detectable limit. Some recent studies present the application of this unique technique to analyze favipiravir in human plasma and breast milk^10,11^ pioglitazone, repaglinide, and nateglinide in human plasma,^12^ vitamin B12 in saliva,^13^ nonsteroidal anti-inflammatory drugs in total blood,^10^ venlafaxine, paroxetine, fluoxetine, amitriptyline and clomipramine in human plasma,^14^ and benzylpenicillin, cloxacillin, dicloxacillin, and oxacillin in human plasma samples.^15^

As a result of the globalization of the pharmaceutical market and the establishment of consensus among pharmaceutical companies, the importance of drug safety issues has increased significantly. This has led to ever-increasing demands for ensuring the quality of medicines and ensuring their safety in drug development and marketing. Bioequivalence is the property wherein two drugs with identical active ingredients or two different dosage forms of the same drug possess similar bioavailability and produce the same effect at the site of physiological activity.^16^ The generic medicinal product must, in particular, be therapeutically equivalent and interchangeable with the reference medicinal product.^17^ Testing bioequivalence between an equivalent medicinal product and a suitable reference medicinal product (pharmaceutical equivalent or pharmaceutical alternative) through a pharmacokinetic study with a limited number of volunteers is a way to demonstrate therapeutic equivalence without conducting a clinical trial involving many volunteers. In such a pharmacokinetic study, any statement regarding the safety and efficacy of the test product is based primarily on the measurement of systemic concentrations, assuming that similar plasma concentrations of the active pharmaceutical ingredient (API) and its metabolite will result in similar concentrations at the site of action and therefore a similar therapeutic outcome.^18,19^

In any study conducted in the laboratory, if the pharmaceutical analysis part is not a routine application but just a one-time operation to obtain the significant data, in such a case, time-consuming procedures, multiple steps requiring high precision, reproducibility of analysis results between days, and cost may not be the priority of researchers. However, in pharmacokinetic applications, including bioequivalence studies, many samples are analyzed regularly. To manage some scenarios, including transferring the samples to the legal authorities, the bioequivalence centers must be forced to comply with regulations to ensure the reliability of the data.^20,21^

In this study, levofloxacin (LEV) was selected as a model molecule, and a liquid chromatography–mass spectrometry (LC-MS)-based analytical method was developed for the quantification of LEV in rabbit blood plasma based on intravenous (IV) administration. Although LC-MS methods have been reported to analyze LEV from plasma in previous studies, this study is the first to analyze the FPSE of LEV from blood plasma.

What makes this study different from the previous studies is not only the usage of FPSE as a microextraction device but also the use of FPSE membrane as a sample storage and transfer device for short-term applications, where, in routine applications, samples need to be protected and transferred using a cold chain to keep samples stable.

Extraction was performed using a volume as low as 10 μL of plasma samples. After the treatment of LEV plasma samples onto FPSE, they were kept dried and stored in the refrigerator (2–8 °C), at ambient temperature (20 ± 5 °C), and in the stability cabinet (40 °C, 75% humidity), respectively. Three different conditions—Eppendorf tubes, plastic containers, and straw paper envelopes—were used to keep the samples inside. The samples were protected from daylight for at least 1 week in the mentioned conditions. LEV assays were analyzed using the developed LC-MS method, and the stability of the samples was evaluated. The proposed methodology was adapted to pharmacokinetic applications, and the results were compared with those obtained using routine protein precipitation and LC-MS quantification.

## 2 Materials and Methods

### 2.1 Chemicals and Standard Stock Solutions

Acetonitrile and methanol were purchased from J.T. Baker (Pennsylvania). Formic acid was obtained from Merck (Darmstadt, Germany). LEV and Ciprofloxacin (IS) standards were provided by Drogsan Pharmaceuticals R&D Laboratory (Ankara, Turkiye). LEV stock solution (400 μg mL^–1^): 40 mg of LEV standard was weighed into a 100 mL volumetric flask. 50 mL of acetonitrile was added and left in an ultrasonic bath for 15 min. After the solution is brought to room temperature, its volume is made up using acetonitrile. IS stock solution (200 μg mL^–1^): 20 mg of Ciprofloxacin HCl was weighed into a 100 mL volumetric flask. 50 mL of dilution solution was added and left in an ultrasonic bath for 15 min. After the solution is brought to room temperature, its volume is completed with a dilution solution. Dilution solution [formic acid 0.1% (v/v)]: 1 mL of formic acid was added to 1000 mL of pure water and mixed.

### 2.2 Standard Solutions

0.005 (LLOQ; lower limit of quantitation), 0.008, 0.010, 0.100, 0.200, 0.500, 1.000 μg mL^–1^ LEV solutions including 0.2 μg mL^–1^ were obtained by dissolving appropriate amounts of LEV stock solution (400 μg mL^–1^) and IS stock solution (200 μg mL^–1^) in dilution solution. Calibration range is defined by LLOQ, which is the lowest calibration standard.

### 2.3 Fabric Phase Sorptive Extraction of LEV

**Figure 1:**
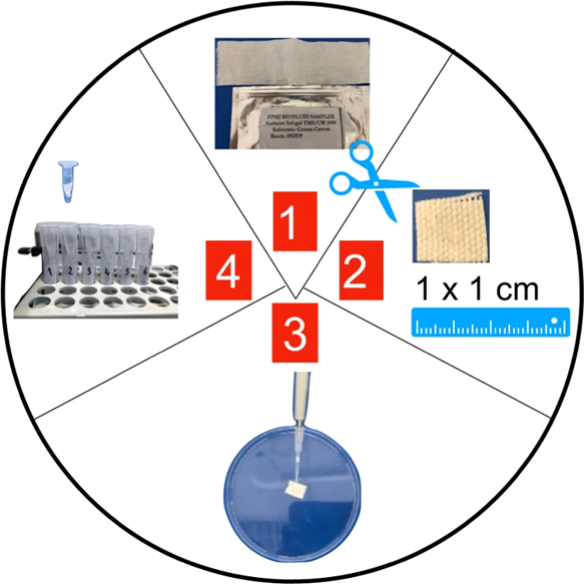
A schematic view of the treatment and extraction of LEV using FPSE.

FPSE biofluid sampler (Sorbent: Sol–gel TMS/CW 20M, Substrate: Cotton Canvas Batch:092519) was used. This biofluid sampler was produced by chemically adding hydrophobic alkyl chains [–O-(−CH_2_–CH_2_–O−)*_n_*–H] to the silanol groups (the chemical structure of the sorbent is given in the Supporting File). The fabric was measured with a ruler and cut with scissors to be 1 cm × 1 cm in size. 10 μL of (a) LEV-containing standard solutions (for recovery studies), (b) LEV-spiked plasma samples (for recovery and stability studies), and (c) LEV-containing plasma samples (for pharmacokinetic studies) were applied with the help of a micropipette. The membranes were dried at room temperature, a 1 cm × 1 cm FPSE membrane was placed in an Eppendorf tube, and 1000 μL of dilution solution was added. The samples were shaken using a Heidolph Unimax 1010 (Schwabach, Germany) shaker device for 60 min. Afterward, the samples were centrifuged at 4100 rpm for 10 min using the Nüve/NF400 brand device. The resulting clear solutions were transferred to a device-specific vial to be analyzed on the LC-MS device. The workflow of this simple sampling and sample preparation protocol is schematized in Figure 1.

### 2.4 LEV Analysis Using Protein Precipitation

2250 μL of methanol was added to 250 μL of plasma samples and vortexed and then centrifuged, and 50 μL of the supernatant was taken, and 50 μL of IS at a concentration of 4 μg/mL was added and mixed with 900 μL of dilution solution. It was then injected into LC-MS under the analysis conditions.

### 2.5 FPSE Stability Studies

**Figure 2:**
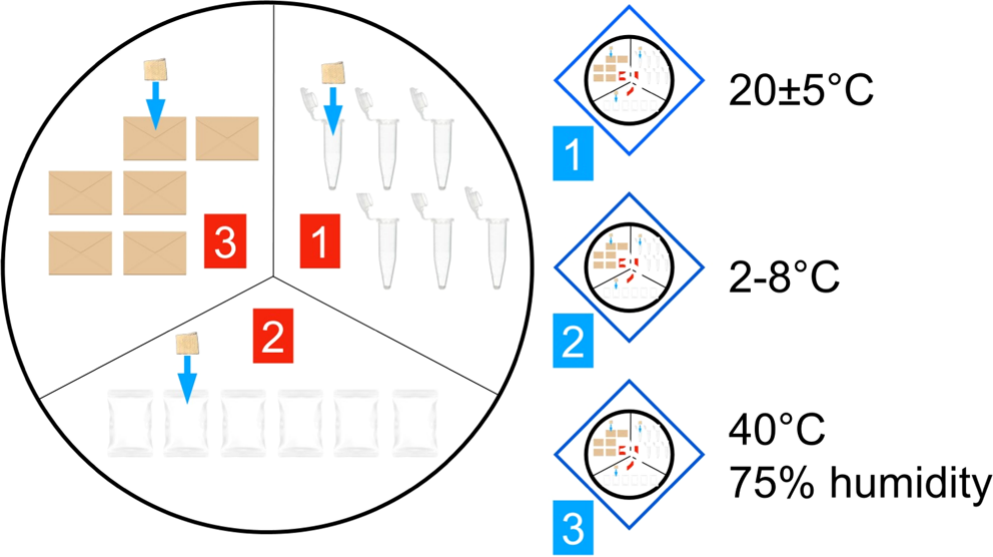
Schematic view of the stability studies performed using plasma spiked FPSE fabrics.

Three different scenarios were simulated for the use of FPSE for the storage and transfer of plasma samples containing LEV. For this purpose, a total of nine different conditions were tested, including three different temperatures [refrigerator (2–8 °C), ambient temperature (20 ± 5 °C), and stability cabinet (40 °C, 75% humidity)] and three different storage conditions (Eppendorf tubes, plastic containers, and straw paper envelopes). The stability of LEV (0.1 μg mL^–1^) in plasma samples applied onto FPSE was examined at ambient temperature (20 ± 5 °C) for 1 day and in all short-term conditions for 1 week. 0.1 μg mL^–1^ LEV-spiked plasma samples kept under 9 different conditions were analyzed as six replicates. Figure 2 presents a schematic preview of the studies applied for stability studies.

### 2.6 Pharmacokinetic Studies

A New Zealand male rabbit was used for the experiment, which was performed with the approval of the NESA Animal Experiments Local Ethics Committee (Document Date and Number: 02/08/2023–014). In the study carried out using a New Zealand White Rabbit, the ear of the rabbit was wetted with warm water to make the veins more visible. Subsequently, LEV (500 mg/100 mL of drug at a dose of 5 mg/kg) was administered IV. 500 μL blood samples were taken at 5, 15, 30, 45, 60, 90, 120, 240, 340, 480, 600, 1440, and 2880 min. The sample was placed in a tube containing lithium heparin and centrifuged at 10, 000 rpm for 10 min. Then, the plasma part was placed in separate vials and stored at −80 °C until the day of the experiments. Besides extraction of LEV using FPSE, protein precipitation was applied to obtain a clear supernatant to be injected.

### 2.7 Analytical Method Validation

Analytical method validation was performed based on ICH M10 bioanalytical method validation and study sample analysis guidelines.^22^ For the linearity study, 7 different concentrations [0.005 (LLOQ), 0.008, 0.010, 0.100, 0.200, 0.500, and 1.000 (ULOQ) μg mL^–1^] were prepared (*n* = 6), and these solutions were analyzed in the LC-MS system; the ratio of the LEV peak area to the IS peak area was plotted against the concentration, and the linearity of the LC-MS method was evaluated. The recommendation that the relative standard deviation (RSD%) value of the value obtained for each concentration should be less than 15 was considered. In selectivity studies, blank plasma solutions were introduced into the system, and the criterion of not interfering with any other substance during the retention times of LEV and IS was considered. Specificity studies must show that the analyte peak is separated from all other components (dilution solution, mobile phase, IS). While examining the matrix effect, accuracy was studied at LLOQ and ULOQ levels in three repetitions. The evaluation was performed based on the criterion that the nominal concentration to be obtained was within ±15%. In examining the carry-over effect, the dilution solution is analyzed immediately after the LLOQ injection, and the highest point of the calibration curve for LEV is analyzed in the system. If a peak is detected in the dilution solution chromatogram at the same retention time as the analyte, this value should not exceed 20% of the LLOQ. Four points, including LLOQ and ULOQ, were selected and injected 5 times each to monitor the injection repeatability. In precision and accuracy studies, LEV samples spiked into plasma at four different concentration [0.005 (LLOQ), 0.015 (3xLLOQ), 0.048, and 0.727 μg mL^–1^] values were extracted and analyzed according to the FPSE extraction procedure, and the RSD% and recovery% values of the analysis results were examined.

### 2.8 Chromatographic Conditions

Separations were carried out on an ACE C18 LC Column (150 mm × 4.6 mm, 5 μm) at 25 °C. The flow rate was 0.5 mL min^–1^ while using isocratic elution with solution A (0.1% formic acid in water): solution B (0.1% formic acid in acetonitrile) (80:20, v/v). The injection volume was 40 μL. The autosampler temperature was set to 5 °C. The run time was determined as 15 min. The retention time of LEV is about 5.4 min. On the other hand, a single quadrupole mass detector was used as the detector with ESI+. Source temperature was set to 120 °C. The desolvation temperature was set to 150 °C. The cone gas flow rate was 50 L h^–1^. Cone voltage and capillary voltage were 10 V and 1 kV. The MS data were recorded in SIR mode (LEV: *m*/*z* 362, IS: *m*/*z* 332).

## 3 Results and Discussion

Under the optimized conditions given in the experimental section, LEV was successfully analyzed in less than 15 min in isocratic elution mode. The single-step extraction used 10 μL of the plasma sample without prior application. An aqueous dilution solvent was used as the extraction solvent, and the samples were placed in an orbital shaker for 60 min. The system suitability results for the peak of LEV are as follows: peak tailing is 1.17, and theoretical plate counts (*N*) is 8533.

### 3.1 Analytical Method Validation

#### 3.1.1 Selectivity and Specificity

**Figure 3:**
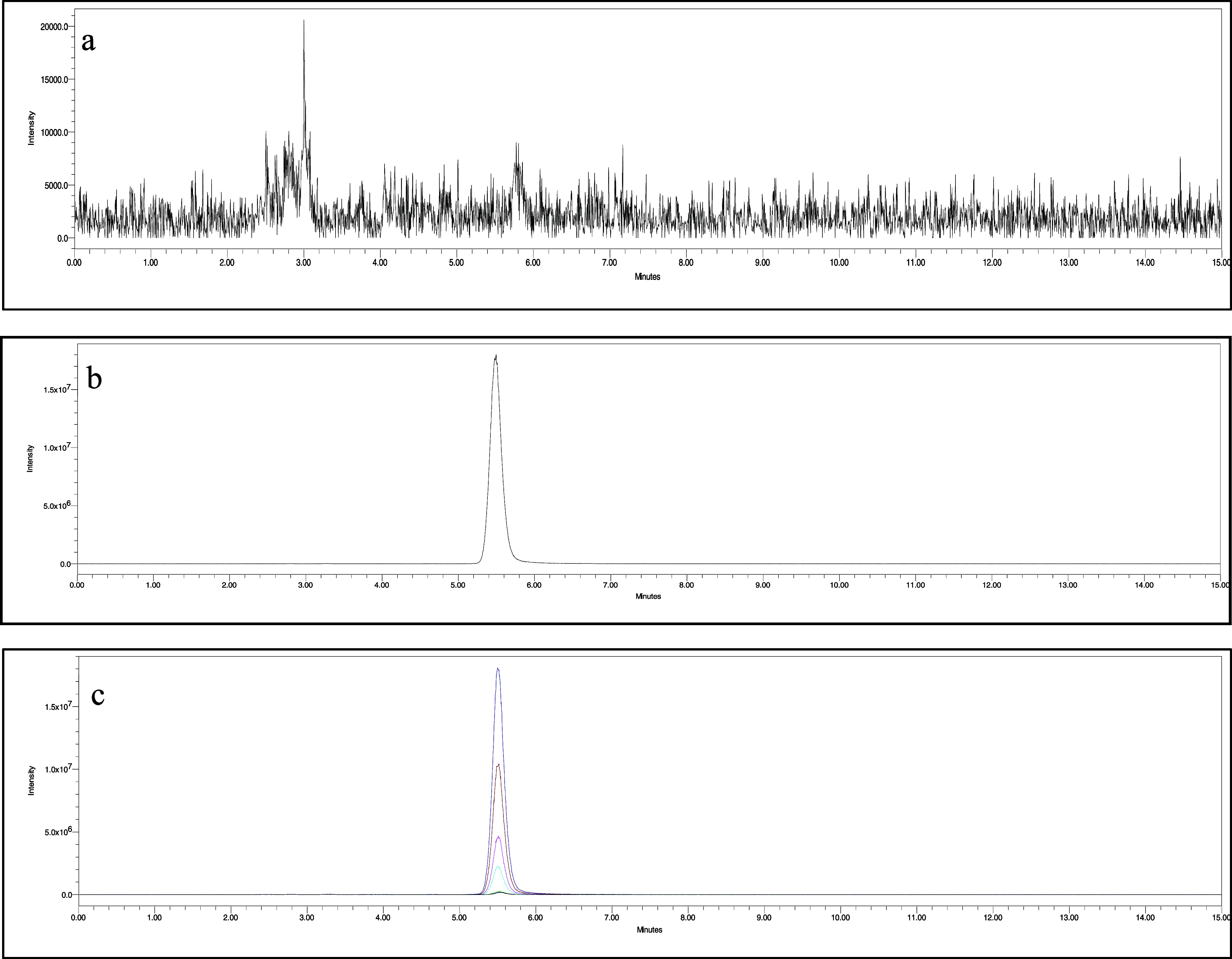
Chromatograms taken under optimum conditions. (a) Extracted blank plasma chromatogram, (b) LEV extracted from spiked plasma samples as 1.0 μg mL^–1^, and (c) LEV standard solutions [0.005, 0.008, 0.010, 0.100, 0.200, 0.500, 1.000 μg mL^–1^].

In this section, we assess the specificity and selectivity of the method employed in our study to ensure the accurate detection and quantification of our target analytes. Specificity refers to the ability of the method to distinguish the target analyte from interfering substances, while selectivity indicates the method’s ability to measure the analyte accurately in the presence of potential confounding factors. The specificity of the developed method was evaluated through a series of tests involving the injection of pure standards and plasma samples with complex matrices. The chromatographic system effectively resolved the target analytes from potential interferences, demonstrating its high specificity. We employed mass spectrometry (MS) as the detection technique to quantify our analytes. The selectivity of the MS method was tested by assessing its response to our target analytes at varying concentration levels. The mass spectrometer exhibited a linear response to changes in analyte concentration, with minimal interference from coeluting compounds, confirming its selectivity. Figure 3 presents the chromatograms taken under the optimum conditions for determining LEV in plasma samples. Results from these studies consistently demonstrated the ability of our method to quantify the target analyte LEV specifically and selectively, even in the complex matrices of plasma.

#### 3.1.2 Linearity

Linear regression analysis was performed on the data obtained from the standard solutions to construct the calibration curve. The calibration curve displayed a strong linear relationship between the concentration of the analyte and the corresponding peak area ratios of analyte and IS (*y* = 3.1413 *x* + 0.0419, *R*^2^ = 0.9966, number of data points: 7, *n* = 6)

#### 3.1.3 LLOD and LLOQ

In this section, we report the determination of the lower limit of detection (LOD) and lower limit of quantification (LOQ) for our LC-MS method. These parameters are critical in assessing the sensitivity and performance limits of the analytical method, providing guidance on the lowest concentration of the target analytes that can be reliably detected and quantified. To establish the LOD and LOQ, a series of standard solutions containing the target analytes were prepared in a solvent matrix that closely resembled the sample matrix. These solutions covered a range of concentrations, including concentrations near or below the expected LOD and LOQ. While the concentration value of the chromatogram in which the signal/noise ratio of the LEV solution is 10.2 for 0.005 μg mL^–1^, the concentration value of the chromatogram in which the signal/noise ratio for 3.0 is 0.002 μg mL^–1^. Under these conditions, the LLOQ and LLOD values of the analytical method were found to be 0.005 and 0.002 μg mL^–1^, respectively.

#### 3.1.4 Precision and Accuracy

The precision and accuracy of the LC-MS method were rigorously evaluated to ensure the reliability and reproducibility of the analytical measurements. LEV samples spiked into plasma at four different concentration (0.005, 0.015, 0.048, 0.727 μg mL^–1^) levels, including the LLOQ value, were extracted using FPSE. Intraday and interday precisions were determined by analyzing a set of replicate samples (*n* = 6) of the same matrix containing known concentrations of the target analytes. Intraday and interday accuracies were assessed by comparing the measured concentrations to the known concentrations of the spiked samples. The results are given in the Supporting Information File, where the RSD% is between 1.0 and 8.6%, and recovery values are higher than 97.2%. Intraday and interday results exhibited low variability and percentage errors, indicating the method’s ability to consistently produce reliable and accurate measurements.

#### 3.1.5 Injection Repeatability

The injection repeatability tests were performed using a representative set of samples [0.005 (LLOQ), 0.008, 0.010, 0.100, 0.200, 0.500, and 1.000 (ULOQ) μg mL^–1^] containing the target analytes at known concentrations. The samples were prepared following the method described in the Sample Preparation section of this paper. These samples were injected within the same day, and the results were considered using the peak area of LEV and the peak area ratio of LEV to IS. Based on our results, the RSD% values of peak areas were 7.2, 3.3, 1.3, 3.2, 3.0, 2.3, and 4.3, where the RSD% values of peak area ratios were 4.7, 3.0, 4.2, 2.8, 1.9, 1.0, and 1.0 for identical concentrations, respectively. These results indicate that the sample analysis system, including the injector and MS detector, consistently delivered precise and reproducible results when an IS was used.

#### 3.1.6 Carry-Over Effect

After the solution prepared at the ULOQ concentration (1.0 μg mL^–1^), which is the highest point of the calibration curve, was read in the system, 0.1% formic acid solution, which is the dilution solution, was read in the system immediately afterward, and the superimposed chromatograms of consecutive injections are shown in the Supporting Information File. The peak area of the dilution solution (blank solution) was 0.08 of the LLOQ value (0.005 μg mL^–1^) and was acceptable.

#### 3.1.7 Robustness

To evaluate the method’s robustness, we deliberately introduced small variations in method parameters while keeping other conditions constant. These variations were chosen to simulate potential sources of minor variation during routine analyses. For this purpose, changes were made in the mobile phase ratio [Solution A: Solution B 70:30 (v/v)] flow rate (0.4 and 0.6 mL min^–1^) and column temperature (20 and 30 °C), and the results were evaluated statistically. Although lowering the column temperature by 20 °C shifted the retention time to a value of around 6 min, when we performed regression analysis with solution injections within the linearity range, the correlation coefficient was found to be 0.998, and the system suitability parameters still met the necessary criteria (*N* > 8000, peak tailing <1.5). When the results were evaluated statistically, the results obtained for 0.005 μg mL^–1^ (LLOQ), 0.1, and 1.0 μg mL^–1^ (ULOQ) values were in integrity with the results obtained under optimized conditions (*p* > 0.05). This shows that the method is robust against column temperature changes. Changing the flow rate to 0.4 and 0.6 mL min^–1^ caused dramatic changes in retention time as expected, and although the system suitability parameters were within acceptable limits, there was a 15% increase in peak areas with a decreasing flow rate and an increase of 15% with an increasing flow rate. A decrease of approximately 15% was detected. Under these conditions, it was observed that the method could produce erroneous results depending on flow rate changes. Changing the mobile phase ratio caused tailing in the peaks (peak tailing > 1.5) and caused a half-decrease in the peak area. In this case, it was concluded that the developed analytical method was only resistant to column temperature changes.

### 3.2 Stability Studies for LEV Extracted from Plasma and Dried on FPSE

LEV-spiked (0.1 μg mL^–1^) plasma samples were applied to FPSE in 10 μL and dried and then stored in a refrigerator (2–8 °C), ambient temperature (20 ± 5 °C), and stability cabinet (40 °C, 75% humidity). They were kept in Eppendorf tubes, plastic containers, and straw paper envelopes. When the samples kept at ambient temperature (20 ± 5 °C) were analyzed after 24 h, a decrease in the amount of LEV was detected, and it was understood that LEV could not remain stable at room temperature even in a dry state on FPSE. Among the three storage conditions, the environment in which LEV remains less stable is plastic containers. A decrease of up to 20% in the total amount could be detected in the first 24 h. When the results are examined, it is seen that sample transfer of real plasma samples containing LEV on FPSE is not possible under room conditions (Table 1). When the stability results obtained for the samples kept at (20 ± 5 °C) after 1 week are examined, it is seen that the amounts decrease compared to the first 24 h, but this decrease is not as rapid as in the first 24 h. This situation can be thought to result from the fact that some LEV on the FPSE is trapped in a relatively protected area, away from moisture, and in a condition resistant to oxidation after it dries due to its adhesion within its pores. LEV, which initially degrades rapidly, is thought to be the part in contact with air and moisture in the top layer of the FPSE. It is already known that LEV cannot remain stable in high temperatures, and excessive moisture can lead to hydrolysis and degradation.^23−26^ Besides temperature and moisture, oxidation is one of the major degradation factors for LEV. A forced degradation study for LEV reported by Dabhi et al. clearly showed that at least 70% of LEV remained stable after 1 h under extreme pH values where acid or base hydrolysis occurred. However, it was not possible to say identical things when it comes to oxidative stress. More than 65% of LEV was degraded when oxidative stress was applied.^27^ In our experimental conditions, LEV could not be kept stable under extreme temperature and humidity (40 °C and 75% humidity). It degraded around 80% within 1 week in such an aggressive condition. When the temperature was kept low (2–8 °C) for 1 week, LEV was stable in Eppendorf tubes (*p*_calculated_ = 0.08, *p*_table_ = 0.05, and *p*_calculated_ > *p*_table_ 0.05). However, the stability cannot be maintained in plastic containers and straw paper envelopes. This situation can be explained by the fact that the amount of oxygen in the Eppendorf tubes is less, and they do not allow air. The straw paper envelope is an environment that is very suitable for breathing. We do not have information about the compatibility of plastic containers, and since they can retain moisture, it is thought that they promote the degradation of LEV due to moisture and oxidation in an environment containing more oxygen.

**Table 1:** Short-Term Stability Results of LEV When Adsorbed onto FPSE and Stored in Different Containers

	1 week			1 day (24 h)
containers	2–8 °C	20 ± 5 °C	40 °C/75% humidity	20 ± 5 °C
Eppendorf tubes	98.5% ± 0.8	85.8% ± 0.5	18.2% ± 1.1	91.8% ± 2.1
plastic containers	82.3% ± 1.4	74.9% ± 0.5	24.5% ± 1.4	81.4% ± 1.0
straw paper envelopes	95.0% ± 1.4	85.1% ± 0.5	18.2% ± 1.3	94.0% ± 2.0

### 3.3 FPSE Extraction for Determination of the Pharmacokinetic Profile of LEV

**Figure 4:**
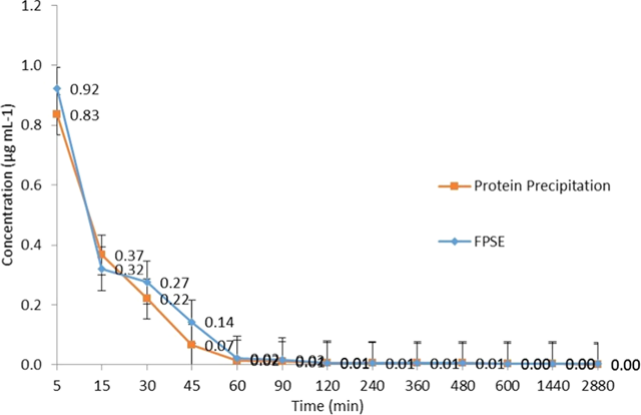
Pharmacokinetic profile of LEV determined using FPSE and protein precipitation.

Blood samples taken at 5, 15, 30, 45, 60, 90, 120, 240, 340, 480, 600, 1440, and 2880 min were separated into two equal parts using Eppendorf tubes. The first part was extracted using FPSE, the second part was simultaneously treated with methanol, and proteins were precipitated, as described in the experimental part. The extracted samples using FPSE and the supernatant of the samples after precipitation of the proteins were injected into LC-MS. The pharmacokinetic profile for LEV is given in Figure 4. When the values obtained after the injection of the solutions obtained by FPSE and protein precipitation techniques were compared statistically, it was seen that the F test defined unequal variance for the fifth, 15th, and 30th minute results. When the test for unequal variances was performed, it was determined that the results at the fifth, 15th, and 30th minutes did not have a statistically significant difference at the 95% confidence level (*p* values are 0.09, 0.20, and 0.06, respectively).

### 3.4 Discussion

The pharmaceutical industry can be defined as one of the most critical industries in the world in terms of production volume, commercial capacity, and social aspects.^5−7^ A drug containing the same active ingredients in the same ratio as the original drug must be pharmaceutically bioequivalent to be defined as generic.^9^ As the generic drug market grows in the world, and in addition to this, as the competition among companies improves, the pressure on R&D and quality control laboratories is increasing with the development of new generic drugs.^10,14^ In addition to generic drugs, the pharmaceutical industry routinely offers new formulations and dosage forms. Different indications of known active substances require the production of different dosage forms to use those active substances for different purposes. In this case, it is important to reveal the pharmacokinetic profiles of the generic drugs, new dosage forms, and formulations. In bioequivalence studies, proving that the distribution of the original and generic drugs in the body is similar within acceptable criteria also challenges preclinical and clinical research centers and laboratories specialized in this field. In preclinical and clinical studies, the laboratory area where biological material is collected and analyzed in human experiments cannot always be in logistically close locations. This situation, which may seem like a minor problem, can be achieved in scientific research by maintaining the cold chain and preparing samples for analysis. However, in routine applications subject to regulations, such as bioequivalence studies, the protocol regarding the transfer of the sample must be carried out by the relevant regulations. In this study, the active substance LEV was selected as a model molecule, and an LC-MS method was developed to prepare samples using the FPSE technique to analyze LEV in blood plasma. Among some other FPSE biofluid samplers coded as Sol–gel TMS/PheTES/CW 20M and Sol–gel TMS/PheTES/APTES/CW 20M, we used the one having nonpolar hydrophobic alkyl chains coded as Sol–gel TMS/CW 20M. As mentioned in the experimental part, this choice allowed us to extract LEV with a higher recovery from blood plasma using a simple aqueous extraction solvent. To improve the recovery of LEV from blood plasma samples, the extraction volume was arranged as 1:100 sample/extraction solvent (v/v), and the elution time was kept as much as possible, and it was 60 min in the shaker. The developed LC-MS method was validated using the ICH M10 bioanalytical method validation guidelines. It was observed that the method could perform LEV analysis in blood plasma within acceptable criteria. The developed method was applied in a pharmacokinetic study for the analysis of LEV, and it was determined that the results were statistically similar to the analysis of the supernatant as a result of the precipitation of proteins with organic solvent, which is an accepted method used in most blood plasma analyzes in routine practice (Figure 4). The recovery value for the developed method is between 97.2 and 103.6% when examined over four different concentration values, including LLOQ. The fact that the FPSE-based extraction and LC-MS method can work seamlessly for pharmacokinetic applications has encouraged us to investigate the feasibility of using direct FPSE in sample transfer protocols. The amount of plasma used in the extraction performed with FPSE is 10 μL. However, when LC-MS publications regarding LEV analysis are examined, it is seen that the minimum amount of plasma used is around 50 μL. Therefore, we tested our prediction that as little as 10 μL of a sample could be applied to FPSE, allowed to dry, and transferred in an Eppendorf tube, plastic container, or straw paper envelope. However, as can be seen from the results (Table 1), we found that it can be stored in an Eppendorf tube at 2–8 °C for a maximum of 1 week. This shows that LEV adsorbed to the FPSE surface and internal pores in blood plasma are still exposed to oxidative stress after drying and cannot remain stable due to interaction with heterogeneous matrix components. What distinguishes the Eppendorf tube from other storage media is that it can carry the LEV on the FPSE phase in a very small volume, without contact with air and away from moisture. This study practically showed the usage of FPSE biofluid sampler as a sample storage and extraction device. By decreasing the steps to prepare samples and using micro amounts, the FPSE biofluid sampler-assisted LC-MS methodology was scored using the online tool of Blue Applicability Grade Index (BAGI) (https://bagi-index.anvil.app), which is proposed as a new metric tool for evaluating the practicality of an analytical method. The BAGI score for our method was 57.5 [the higher the score, the more practical the method (from 25 to 100)].^28^

## 4 Conclusions

The analysis of LEV, which was selected as a model molecule in this study, from blood plasma with high recovery (>95.0%) could be performed by FPSE using a sample volume as small as 10 μL. When LEV extracted by FPSE was analyzed by LC-MS, it was seen that the results of the pharmacokinetic study were statistically compatible with injection-based techniques after precipitation of proteins with organic solvent used in routine applications. Studies on using FPSE as a sample transfer equipment due to applying blood plasma onto FPSE have shown that such a transfer is possible only for a maximum of 1 week under conditions where Eppendorf tubes are used and 2–8 °C is maintained. Ultimately, it has been shown that it is possible to analyze LEV from blood plasma by FPSE and use it in pharmacokinetic applications. For many LEV-like molecules, analysis with FPSE is especially important in conditions where low sample volumes are required. This study is important in terms of using FPSE in pharmacokinetic applications and shows that it can enable sample transportation as a sample carrier system without the need for aggressive cold chains.

## Supporting Information Available

The chemical structure of the FPSE biofluid sampler (sorbent: Sol–gel TMS/CW 20M, substrate: cotton canvas batch: 092519); the chromatogram for IS; the chromatogram showing the carry-over effect; and the data presenting the precision and accuracy of the developed method (PDF)
