# Supplementary material for: Adapting Fabric Phase Sorptive Extraction as an Innovative Multitool for Sample Transfer and Extraction in Pharmacokinetic Analysis Followed by LC-MS Determination of Levofloxacin in Plasma Samples
Source: ACS Omega. 2024 Apr 15;9(17):18995–9002. doi: 10.1021/acsomega.3c09519 (PMC11064165; doi:10.1021/acsomega.3c09519)
Supplement: Supplementary file 1 — ao3c09519_si_001.pdf [file ao3c09519_si_001.pdf]

Adapting Fabric Phase Sorptive Extraction as an Innovative Multitool for Sample Transfer and Extraction in Pharmacokinetic Analysis followed by LC-MS determination of Levofloxacin in Plasma Samples

Yasemin Ekin Dolaksız[1], Mustafa Sinan Kaynak[2], Abuzar Kabir[3*], Kenneth G. Furton[3], Mustafa Çelebier[1*]

1 Hacettepe University, Faculty of Pharmacy, Department of Analytical Chemistry, 06230 Ankara, Turkiye

2 Anadolu University, Faculty of Pharmacy, Department of Pharmaceutical Technology, 26460, Eskişehir, Turkiye

3 International Forensic Research Institute, Department of Chemistry and Biochemistry, Florida International University, 11200 SW 8th St., Miami, FL 33199, USA

This supplementary file includes the chemical structure of FPSE Biofluid Sampler (Sorbent: Sol-gel TMS/CW 20M, Substrate: Cotton Canvas Batch:092519) (1) given in Figure 1 and chromatogram of IS given in Figure 2. Figure 3 presents chromatogram to show carry-over effect. Table 1 presents the data for the precision and accuracy of the developed method.

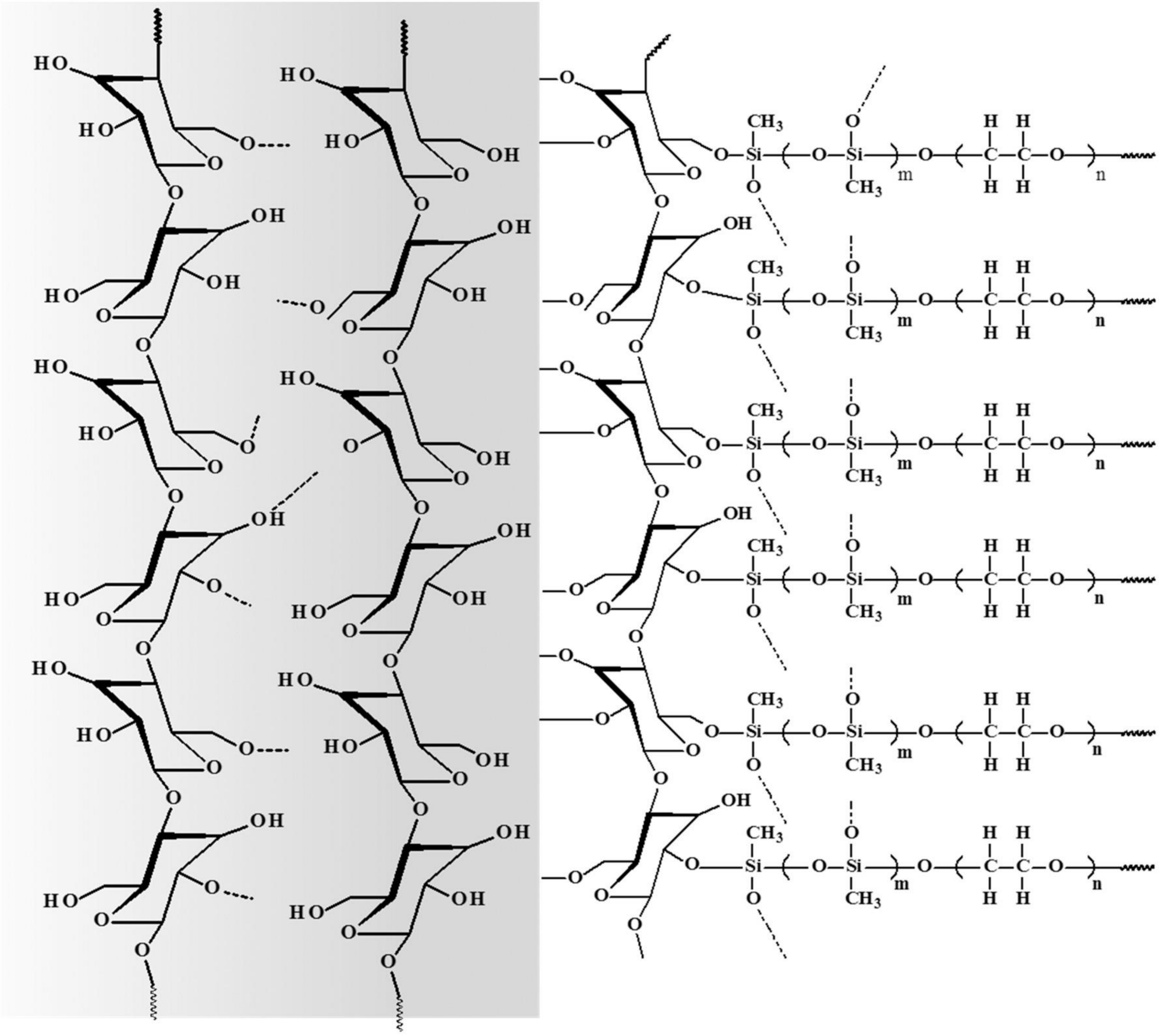

Figure 1. Presentation of sol-gel CW 20M coated FPSE membrane [1]

IS peak is shown in Figure 2.

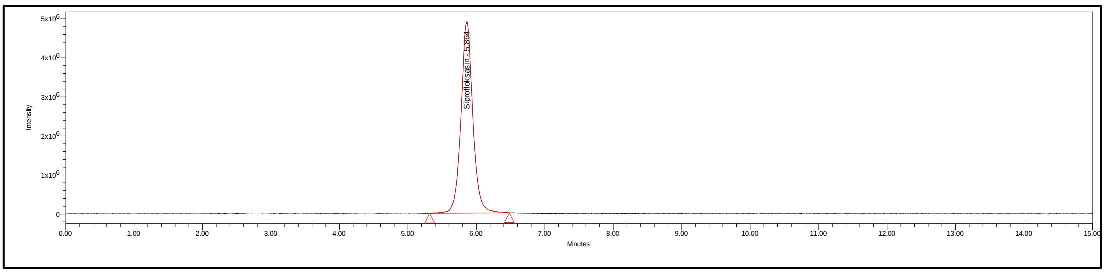

Figure 2: Chromatogram of IS (0.2 μg/mL) solution (m/z:332).

The overlapped chromatograms to show carry-over effect are given in Figure 3.

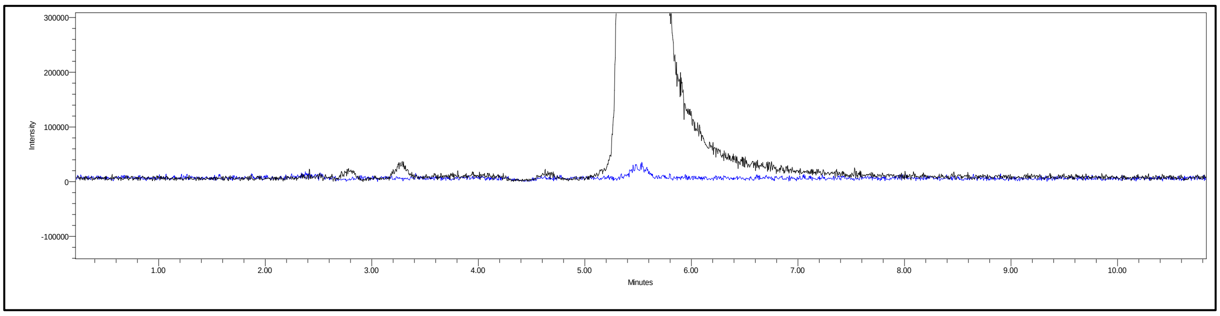

Figure 3. LEV, ULOQ solution chromatogram (black) and dilution solution chromatogram (blue)

Table 1. Precision and accuracy results of the developed method

Sample #	Spiked Concentration (µg mL-1)	Day #1 Determined Concentration (µg mL-1)	Recovery %	Mean %	RSD %	Day #2 Determined Concentration (µg mL-1)	Recovery %	Mean %	RSD %
1	0.0048	0.0045	92.5			0.0046	95.9		
2	0.0048	0.0044	91.3			0.0047	96.7		
3	0.0048	0.0046	94.2	100.1	8.6	0.0051	105.0	101.9	4.5
4	0.0048	0.0053	108.8			0.0050	103.8		
5	0.0048	0.0054	110.7			0.0050	103.2		
6	0.0048	0.0050	102.9			0.0052	107.1		
1	0.0145	0.0140	96.1			0.0134	92.1		
2	0.0145	0.0146	100.6			0.0154	106.3		
3	0.0145	0.0152	104.8	97.2	4.7	0.0151	103.9	97.8	6.3
4	0.0145	0.0136	93.4			0.0142	98.0		
5	0.0145	0.0136	93.9			0.0133	91.3		
6	0.0145	0.0137	94.2			0.0138	95.2		
1	0.0485	0.0475	98.0			0.0524	108.2		
2	0.0485	0.0467	96.4			0.0496	102.3		
3	0.0485	0.0503	103.9	98.4	2.8	0.0491	101.3	103.6	2.3
4	0.0485	0.0473	97.6			0.0498	102.9		
5	0.0485	0.0474	97.8			0.0505	104.2		
6	0.0485	0.0467	96.5			0.0499	103.0		
1	0.7268	0.7154	98.4			0.7218	99.3		
2	0.7268	0.7309	100.6			0.7401	101.8		
3	0.7268	0.7190	98.9	99.1	0.8	0.7395	101.7	101.0	1.0
4	0.7268	0.7197	99.0			0.7350	101.1		
5	0.7268	0.7153	98.4			0.7293	100.4		
6	0.7268	0.7210	99.2			0.7387	101.6		

RSD%: Relative standard deviation

REFERENCES

(1) Tartaglia, A.; Kabir, A.; Ulusoy, S.; Sperandio, E.; Piccolantonio, S.; Ulusoy, H. I.; Furton, K. G.; Locatelli, M. FPSE-HPLC-PDA analysis of seven paraben residues in human whole blood, plasma, and urine. *J Chromatogr B Analyt Technol Biomed Life Sci* **2019**, *1125*, 121707. DOI: 10.1016/j.jchromb.2019.06.034 From NLM.
